# Supplementary material for: Reconciling Mining with the Conservation of Cave Biodiversity: A Quantitative Baseline to Help Establish Conservation Priorities
Source: PLoS One. 2016 Dec 20;11(12):e0168348. doi: 10.1371/journal.pone.0168348 (PMC5173368; doi:10.1371/journal.pone.0168348)
Supplement: S1 Dataset — (ZIP) [file pone.0168348.s002.zip › Taxa/Serra Norte/SN_2013/N5S/Lista N5S-51.pdf]

CAVIDADE N5S-0051

Classe	Ordem	Fam/Outros	Gên/Outros	Espécie	S-U	U-S	Zon.
Annelida	Oligochaeta			jovem	1		E
Arachnida	Acari	Mesostigmata		jovem	X		E
Arachnida	Araneae	Mysmenidae		jovem	X		E
Arachnida	Araneae	Oonopidae		jovem		X	E
Arachnida	Araneae	Pholcidae		jovem	X	X	E
Arachnida	Araneae	Pisauridae	*Architis*	sp.1		1	E
Arachnida	Araneae	Pisauridae	*Thaumasia*	*velox*		1	E
Arachnida	Araneae	Theridiidae	*Theridion*	sp.1		X	E
Arachnida	Araneae	Theridiidae	*Theridion*	sp.2		X	E
Arachnida	Araneae	Theridiidae	*Theridion*	sp.4		X	E
Arachnida	Araneae	Theridiosomatidae	*Plato*	sp.1	X	X	E
Arachnida	Opiliones	Sclerosomatidae	*Prionostema*	sp.1		5	E
Arachnida	Pseudoscorpiones	Chthoniidae	*Pseudochthonius*	sp.1		X	E
Diplopoda	Polydesmida	Chelodesmidae		jovem	1		E
Diplopoda	Polydesmida	Fuhrmannodesmidae		jovem		X	E
Diplopoda	Polydesmida	Pyrgodesmidae		jovem		X	E
Entognatha	Collembola	Cyphoderidae		sp.1	X		E
Entognatha	Collembola	Entomobryidae		sp.1	X		E
Entognatha	Collembola	Entomobryidae		sp.9	X		E
Entognatha	Collembola	Isotomidae		sp.1	X		E
Entognatha	Collembola	Paronellidae		sp.1	X	X	E
Insecta	Blattodea	Blattellidae		sp.1	1	1	E
Insecta	Coleoptera	Staphylinidae	Pselaphinae	sp.7		X	E
Insecta	Coleoptera	Staphylinidae		sp.4		X	E
Insecta	Coleoptera	Staphylinidae		sp.30	X		E
Insecta	Diptera	Cecidomyiidae	Cecidomyiinae	sp.	X		E
Insecta	Diptera	Culicidae	*Culex*	sp.		X	E
Insecta	Diptera	Psychodidae	*Pericoma*	sp.		X	E
Insecta	Diptera	Tipulidae	Tipulinae	sp.	X	X	E
Insecta	Heteroptera	Dipsocoroidea		sp.	X		E
Insecta	Hymenoptera	Formicidae	*Acanthognathus*	sp.1		X	E
Insecta	Hymenoptera	Formicidae	*Hypoponera*	sp.1	X		E
Insecta	Hymenoptera	Formicidae	*Hypoponera*	sp.2	X		E
Insecta	Hymenoptera	Formicidae	*Paratrechina*	sp.1	X		E
Insecta	Hymenoptera	Formicidae	*Pheidole*	sp.4	X		E
Insecta	Isoptera	Termitidae	*Nasutitermes*	sp.	X	X	E
Insecta	Orthoptera	Phalangopsidae	*Paraclodes*	jovem		10	E
Insecta	Thysanura	Ateluridae		jovem	X		E
Amphibia	Anura			sp.		1	E
